# “I’m truly free from my eating disorder”: Emerging adults’ experiences of FREED, an early intervention service model and care pathway for eating disorders

**DOI:** 10.1186/s40337-020-00354-9

**Published:** 2021-01-06

**Authors:** Rachel Potterton, Amelia Austin, Michaela Flynn, Karina Allen, Vanessa Lawrence, Victoria Mountford, Danielle Glennon, Nina Grant, Amy Brown, Mary Franklin-Smith, Monique Schelhase, William Rhys Jones, Gabrielle Brady, Nicole Nunes, Frances Connan, Kate Mahony, Lucy Serpell, Ulrike Schmidt

**Affiliations:** 1 Institute of Psychiatry, Psychology and Neuroscience, King’s College London, London, UK; 2 South London and Maudsley NHS Foundation Trust, London, UK; 3 Maudsley Health, Abu Dhabi, UAE; 4 Sussex Partnership NHS Foundation Trust, Brighton, UK; 5 Leeds and York Partnership NHS Trust, Leeds, UK; 6 Central and North West London NHS Foundation Trust, London, UK; 7 North East London NHS Foundation Trust, London, UK; 8 Division of Psychology and Language Sciences, University College London, London, UK

**Keywords:** Eating disorders, Anorexia nervosa, Bulimia nervosa, Binge eating disorder, Early intervention

## Abstract

**Background:**

Eating disorders (EDs) typically start during adolescence or emerging adulthood, periods of intense biopsychosocial development. FREED (First Episode Rapid Early Intervention for EDs) is a service model and care pathway providing rapid access to developmentally-informed care for emerging adults with EDs. FREED is associated with reduced duration of untreated eating disorder and improved clinical outcomes, but patients’ experiences of treatment have yet to be assessed.

**Objective:**

This study aimed to assess emerging adults’ experiences of receiving treatment through FREED.

**Method:**

This study triangulated qualitative data on participants’ experiences of FREED treatment from questionnaires and semi-structured interviews. Participants were 106 emerging adults (aged 16–25; illness duration < 3 yrs) (questionnaire only = 92; interview only = 6; both = 8). Data were analysed thematically.

**Results:**

Most participants reported psychological and behavioural changes over the course of treatment (e.g. reduction in symptoms; increased acceptance and understanding of difficulties). Participants identified five beneficial characteristics of FREED treatment: i) rapid access to treatment; ii) knowledgeable and concerned clinicians; iii) focusing on life beyond the eating disorder; iv) building a support network; v) becoming your own therapist.

**Conclusion:**

This study provides further supports for the implementation of early intervention and developmentally-informed care for EDs. Future service model development should include efforts to increase early help-seeking.

## Plain English Summary

FREED is a service model and care pathway providing rapid access to developmentally-informed care for emerging adults with EDs in the United Kingdom (UK). Whilst previous studies have demonstrated FREED’s effectiveness in reducing ED symptoms, it is also vitally important to also explore patients’ experiences of receiving treatment through this service. To this end, we interviewed and / or collected questionnaire data from 106 emerging adults who had received treatment through FREED. The vast majority of participants reported that treatment had been helpful, and that they had experienced reductions in their symptoms and / or increased acceptance and understanding of their ED. They identified several aspects of treatment that were particularly helpful. Emerging adults reported that treatment had been provided rapidly and the clinicians were knowledgeable and concerned about their ED. Additionally, participants liked that treatment had focused on life beyond their ED and had helped them build a support network and become their own therapist. This study provides further evidence that service models such as FREED should be implemented widely.

## Introduction

Incidence and prevalence of eating disorders (EDs) peak during late adolescence and emerging adulthood, periods of intense psychosocial development [1–6]. Mean age of onset for anorexia nervosa (AN), bulimia nervosa (BN) and other specified feeding and eating disorders (OSFED) is between 15 and 19 years, whilst binge eating disorder (BED) typically occurs slightly later, between 23 and 24 years [1, 2, 4, 7]. Approximately 14% of female and 4% of male university students (18–22 years of age) screen positive for clinically significant EDs, compared to 12-month prevalence estimates amongst the general population of 2.2% for women and 0.7% for men [6, 8].

Timely and effective ED treatment can prevent derailment of development, yet emerging adults’ treatment needs are typically less well met than those of both adolescents and adults [9]. Emerging adults tend to present to specialist ED services with longer duration of untreated ED than adolescents and report less positive treatment outcomes and more negative treatment experiences [10–13]. There are likely several contributing factors to such shortfalls in existing services. In the UK and many other countries, patients receiving ED treatment are required to transition from child and adolescent ED services (CAEDS) to adult ED services (AEDS) at or around 18 years of age [14]. Many young people fall in the gap i.e. are discharged from CAEDs and choose not to seek a referral to AEDS from their GP [15, 16]. Additionally, in the UK statutory wait-time targets do not apply to adult services, and individuals aged 18 years or over wait longer than those aged under 18 years for treatment (NHS Royal College of [17–19]). Even when treatment is received, AEDS can present difficulties for emerging adults [10, 20, 21]. Incompatibility between the distinctive developmental needs of emerging adults and the culture of adult services may contribute to reluctance to access, dissatisfaction, disengagement, and poor clinical outcomes [22]. This incompatibility is particularly relevant to ED services, as there is a clear shift in treatment philosophy— primarily relating to how personal responsibility is understood and managed—in services for under 18s compared to ED services for 18 years and over [23].

First Episode Rapid Early Intervention for Eating Disorders (FREED) was developed as a service model and care package for 16 to 25-year-olds presenting for their first specialist treatment (“first-episode”) with an ED of less than 3 years duration (“recent-onset”) [24]. FREED aims to reduce the length of time between treatment-seeking and receipt of specialist evidence-based treatment, with most patients starting treatment within 4 weeks of referral to the service. FREED also adapts said treatment to the specific developmental needs of emerging adults, for instance by taking a flexible approach to appointment scheduling, increasing involvement of family/friends, and focusing on managing transitions (e.g. to university) [25]. The key characteristics of FREED compared to treatment as usual are outlined in more detail in Table 1.

**Table 1:** Comparison of FREED service model with treatment as usual (TAU) as delivered before the introduction of FREED (adapted from Fukutomi et al., [26])

Characteristic	Service Model	
	FREED	TAU
Target group	Prioritisation of patients aged 16–25 years old with duration of ED of less than 3 years	No prioritisation according to illness stage Priority determined by diagnosis and severity
Referral and engagement	Person-centred, user-friendly, flexible approach reaching out to young people and families	Barriers to access seen as useful gatekeeping / test of patient motivation
	Actively remove barriers to access	Initial appointment communicated via letter
	Engagement call within 48 h of referral from FREED clinician	Patient’s responsibility to contact service prior to assessment
	Multiple methods of contact (e.g. text; emails)	Strict discharge policy if not engaging
	Flexible approach to initial and subsequent appointments (e.g. accommodating cancellations)	
Waiting times	Target of 2 weeks from referral to assessment and 4 weeks from referral to treatment	Statutory waiting time targets
Assessment	Assessment of biopsychosocial needs, including focus on young person’s strengths and priorities	Assessment of biopsychosocial needs Assessment is separate from treatment
	Explore social media use as potential illness maintaining factor	Patient prepared to wait between assessment and treatment, focus on staying safe during this time
	Psychoeducation using personal feedback and information about malleable changes to brain, body and behaviour to encourage early action on change	Limited psychoeducation at assessment Variable involvement of family and friends
	Instil a sense of hope for recovery and at the same time of urgency of action to make changes now (e.g. through goal setting)	
	Assessment is seen as part of treatment	
	Active involvement of family and friends	
Treatment	Evidence-based psychological therapy tailored to stage of illness and emerging adulthood	One-size-fits-all; standard packages of evidence-based treatment determined by diagnosis and severity
	Early dietitian involvement with focus on nutritional change	Medical and dietetic input when necessary
	Emphasis on transitions (e.g. moving to university) with flexible, supportive transition arrangements to provide a safety net. If necessary, continuation of treatment via distance methods (e.g. email, skype) with joint management arrangements with university-based services	Variable focus on nutritional change. Variable family involvement. Variable use of technology. Discharge to other services at transition of care
	Encourage joint sessions (e.g. with a family member)	

FREED’s effectiveness and scalability has been evaluated in a pilot study and in a larger scale study (FREED-Up), both using a similar pre-post quasi-experimental design comparing patients receiving FREED with similar patients seen in the same service(s) previously [26–29]. The single-centre pilot study found that FREED patients had a shorter duration of untreated ED and significantly better treatment uptake than controls [27]. Additionally, FREED anorexia nervosa patients (AN) showed significantly greater clinical improvement and reduced need for intensive additional treatment (inpatient, day-patient) up to 2 years later [26, 28]. The multi-centre FREED-Up study replicated these findings [29, 30].

Alongside quantitatively-based evaluation, it is vitally important to consider patients’ experiences of treatment process and outcomes [31, 32]. Studies of treatment experience can provide insights into the “active ingredients” to achieve change and inform future improvement in service delivery and treatment, as well as assessing fidelity of implementation [31]. Qualitative methodologies are particularly well-suited to understanding phenomena from the individual’s perspective, and are therefore well-placed to explore emerging adults’ experiences of FREED [9]. This is the first study to use qualitative methodologies to explore patients’ views of FREED, and indeed any ED early intervention service adapted to emerging adults’ developmental needs.

### Study aim

To assess emerging adults’ experiences of receiving treatment for their ED through FREED, a service model and care pathway providing rapid access to developmentally-informed care for emerging adults with first-episode, recent-onset EDs.

## Method

### Design

This research was embedded within the FREED-Up study, which was granted approval by the relevant ethics committees. It used a qualitative design, informed by a critical realist philosophical framework [33, 34]. Critical realism occupies a middle-ground between positivist and constructivist paradigms and is therefore interested both in empirical descriptions of phenomena (e.g. patients’ descriptions of their treatment experiences), and in considering how the broader social context may impinges on and construct those descriptions. Critical realism is particularly well-suited to studies related to clinical interventions, as increased understanding of both experiences and their construction can suggest targets for intervention development. Data from semi-structured interviews were triangulated with questionnaire-based data to develop a comprehensive understanding of patient experiences [35].

### Participants

Participants for this research were drawn from the FREED-Up study, which focused on the continued evaluation of the effectiveness and scalability of the FREED service model (see [25, 29] for further details). The FREED-Up study included 278 emerging adults (18–25 years old) with first-episode, recent-onset (< 3 years) (as ascertained by a structured onset interview) DSM-5 EDs (AN, BN, BED or OSFED) who presented for treatment at four specialist ED services in England between 2016 and 2018. The present study specifically focused on exploring emerging adults’ experiences of receiving treatment through FREED.

### Procedure

#### Questionnaires

All FREED-UP study participants who received the FREED intervention (*N* = 278) completed detailed online questionnaires at 3, 6, and 12 months post-entry to the study. As part of these questionnaires, participants were asked optionally at 6 and 12 months “If you would like to share something about your experience with the FREED early intervention service please feel free to write this below. If not please move onto the next section.” 100 participants (36%) responded at both or either time-points.

#### Semi-structured interviews

Selected FREED-Up study participants were informed of the opportunity to participate in an optional interview by phone or email by one researcher (RP). Potential participants were told that the study was about their experiences of getting help and receiving treatment for their ED. No time-limit was placed on consideration of participation. A purposive sampling strategy was used; this involves identification and selection of participants who are likely to be especially knowledgeable or provide diverse perspectives on the phenomenon of interest [36]. In practice, the researcher accessed existing FREED-Up demographic and clinical data and approached participants with characteristics of putative relevance to the research question (e.g. age; diagnosis; gender, ethnicity; living situation, geographic location). Participants were made aware that participation was voluntary, and their decision to participate (or not) would not affect their care or treatment in any way. The researcher also emphasised that both positive and negative experiences were of interest. No monetary incentive was offered for participation. Estimated sample size was informed by the approximate number of participants required in previous comparable studies of emerging adults’ ED care experiences (i.e. between 15 and 20 participants) [10, 37]. However, recruitment continued until saturation; that is, until the themes devised in concurrent data analysis had been fully explored and new data were easily accommodated within them. In total, 45 emerging adults were approached to participate to reach the final sample size of 14 (31%).

Semi-structured interviews were conducted on a one-to-one basis by RP (a PhD researcher with a psychology background) either over the phone (71%) or in person. RP was not involved in treatment of study participants and had no prior relationship to participants. Care was taken to establish rapport and promote open discussion over the phone (e.g. ensuring the participant was in a private location). Interviews were structured around a topic guide, which was developed by RP. The topic guide was informed by evidence-based conceptualisations of FREED and emerging adulthood, and focused on exploring patients’ experiences of their treatment and its impact (see Appendix for interview topic guide). The topic guide was used flexibly and revised iteratively. The average length of the interview was 33 min. All interview audio was recorded and transcribed verbatim. All identifying information was removed at point of transcription.

### Data analysis

Quantitative data was analysed using SPSS software, version 26. Qualitative data from both questionnaires and semi-structured interviews were analysed collectively. Data analysis was undertaken concurrently with data collection using NVivo version 10. Data analysis used the six steps of thematic analysis [38](see Table 2). Consistent with the critical realist framework, the study used a primarily deductive yet flexible coding process. Coding was therefore informed by existing theory and literature on FREED and emerging adults’ experiences of ED treatment [33]. For example, it was expected that participants would report positive experiences of the short waiting-times characteristic of FREED and this was reflected in the initial coding frame. However, coding was not constrained by such expectations, and codes were deleted, reconceptualised, and added as required. Steps three, four and five of the thematic analysis process focused on exploring latent themes i.e. underlying ideas, assumptions and conceptualisations which shape or inform emerging adults’ descriptions of their treatment experiences [33, 38]. For instance, care was taken to consider the ways in which broader societal discourses about EDs impinged on and constructed participants’ descriptions. The dataset was repeatedly reviewed and discussed by the authors to ensure the emerging themes fit with the original data. Researchers also reflected upon their own personal and professional perspectives to increase awareness of/minimise how these beliefs shaped data analysis.

**Table 2:** Steps of thematic analysis

Step	Process
1. Familiarization with data	Transcribe; read and re-read data-set; note down initial ideas
2. Generation of initial codes	Code data-set
3. Search for themes	Collate codes into potential themes
4. Review themes	Check themes against coded extracts and the entire data set; generate a thematic map
5. Define and name themes	Refine the specifics of each theme; generate clear names and definitions
6. Write up	Select and analyse quotations; write a report of the analysis

## Results

### Participant characteristics

The final study sample consisted of 106 participants (questionnaire = 92; interview = 6; both questionnaire and interview = 8) (see Table 3 for summary of participant characteristics, compared to those who did not provide qualitative data). All participants were emerging adults (16–26 years old) with first-episode, recent-onset (< 3 years) DSM-5 EDs (AN, BN, BED or OSFED), and had received treatment through FREED at four specialist ED services in England between 2016 and 2018.

**Table 3:** Participant (and non-participant) demographics at first specialist contact

	FREED-UP participants with experience data (interview and/or questionnaire) (*N* = 106)	FREED-Up participants with experience data (interview) (*N* = 14)	FREED-Up participants with no experience data (*N* = 172)
Age (M ± SD)	20.75 ± 2.56*	20.86 ± 1.99	19.84 ± 2.20*
DUED (M ± SD)	18.62 ± 10.69	21.50 ± 10.55	17.36 ± 10.18
BMI (M ± SD)	20.06 ± 4.63	18.87 ± 3.23	20.11 ± 4.33
EDE-Q score (M ± SD)	4.16 ± 1.10	4.53 ± 0.72*	4.03 ± 1.28*
Gender (% female)	92.5%	93%	93.6%
ED Diagnosis			
AN	38.7%	35.7%	44.2%
BN	31.1%	35.7%	22.7%
BED	0.9%	7.1%	1.2%
OSFED	29.2%	21.4%	32.0%
Ethnicity			
White	71.7%	71.4%	61.0%
Asian	6.6%	0%	11.6%
Black	1.9%	0%	5.2%
Mixed	4.7%	14.3%	8.7%
Other / Unspecified	9.4%	14.3%	10.3%
Occupation			
Student	58.3%	50.0%	67.9%
Employed	35.9%	50.0%	20.8%
Unemployed	5.8%	0.0%	11.3%
Living Situation			
With family	42.5%	35.7%	58.7%
With friends / in halls	35.8%	50.0%	27.9%
With partner	5.7%	0.0%	4.1%
Alone	4.7%	0.0%	5.2%
Other / Unspecified	11.3%	14.3%	4.1%
Treatment			
Completion rate	84.9%	92.9%	57.6%
No. of sessions (M ± SD)	23.27 ± 12.87*	24.71 ± 10.36*	15.77 ± 11.63*

Asterisk indicates that these figures were significantly different at *p* < 0.05

*Abbreviations*: *AN* anorexia nervosa, *BED* binge eating disorders, *BMI* body mass index, *BN* bulimia nervosa, *DUED* duration of untreated eating disorder, *EDE-Q* Eating Disorder Examination Questionnaire, *M* mean, *OSFED* other specified feeding and eating disorder, *SD* standard deviation

### Thematic analysis

**Fig. 1:**
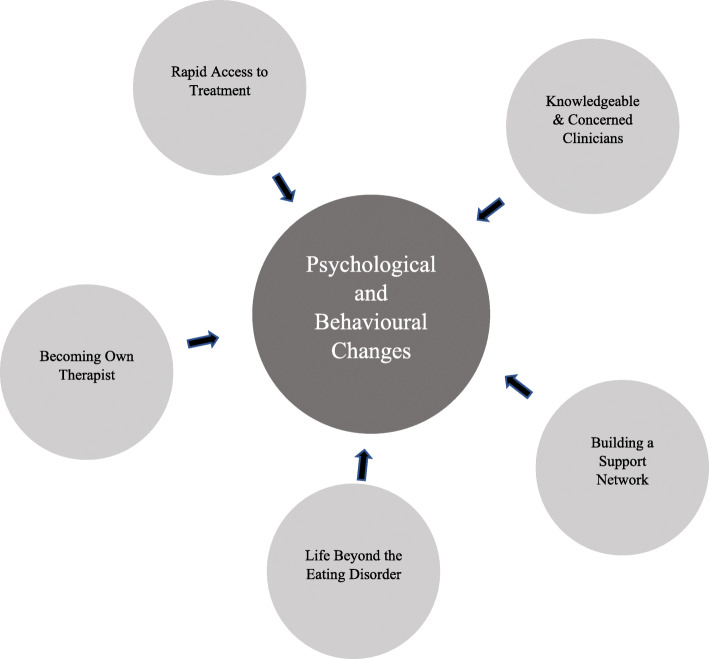
Thematic map

One theme regarding psychological and behavioural changes which the participants had noticed and attributed to the treatment process was identified. Additionally, five themes relating to features of the treatment process were identified. A thematic map is presented in Fig. 1. The data were examined for differences according to participant characteristics (e.g. diagnosis; stage of treatment, data collection time-point [6 months vs. 12 months]), but these did not appear to be associated with differences in the themes identified. Data collection method (interview vs. questionnaire) did appear to be associated with themes identified, such that questionnaire responses tended to relate to treatment impact (i.e. theme 1), whereas the theme related to treatment process was largely derived from the interview data.

#### Psychological and behavioural change

The majority of participants (~ 75% of those providing questionnaire data) reported noticeable psychological and behavioural changes over the course of treatment. All such changes were positively-framed; no participants reported negative treatment-related change. About one-third of participants described dramatic changes (e.g. describing themselves as “recovered”, or treatment as “life-changing”).

*“Comparing myself now to myself at the beginning of [treatment] is like night and day.” (*James, questionnaire)

Other participants were positive about treatment, but more hesitant about the progress they had made. They expressed beliefs that treatment had been an important step in an ongoing journey towards recovery.

*“I may not be completely healed… but I don't feel like the ED is controlling me anymore.”* (Gaia, questionnaire)

*“I have so far to go still but I have also come so far.”* (Jenny, questionnaire)

A small minority (~ 3%) of participants believed treatment was not associated with change but did not tend to elaborate on their beliefs.

*“Although I am grateful to have received treatment quickly, I don't feel that it has been helpful so far.”* (Amelia, questionnaire)

Some of those reporting changes elaborated on the specific nature of these. About 10% of those providing questionnaire data explicitly commented upon reduction of symptoms (e.g. preoccupation with weight and shape, bingeing, purging, restriction) or related improvements in their physical health over the course of treatment.

*“I look back at the last 28 days and how little my eating disorder behaviours have shown up. I have not purged once, and binge-eating thoughts take up a lot less of my time.”* (Luke, questionnaire)

More commonly (about one-quarter of those providing questionnaire data), participants commented on how treatment had changed their perspective on their ED. Many participants described accepting that they had an ED, and increased understanding of their difficulties.

*“I'm finally coming to terms with having an eating disorder.”* (Zira, questionnaire).

*“I have learned lots about my condition.”* (Lydia, questionnaire)

Many participants described how therapy helped them understand their ED as a coping mechanism for stress and difficult emotions. This enabled them to adopt more adaptive strategies when struggling.

*“I have gained skills that I can sometimes apply to cope with difficult situations.”* (Briana, questionnaire)

Some participants (~ 15%) described how treatment helped them understand that their ED was not who they were. Participants described how treatment helped them build an identity separate from their ED.

*“[treatment] helped me to see that, although my eating disorder had infiltrated into all aspects of my life, it wasn't who I was.”* (Amy, questionnaire)

Relatedly, several participants described feeling freer, and more able to be themselves.

*“It's such a relief to wake up in the morning not thinking about it, or throughout the day and at night. I have to be careful not to get too obsessive, but I do feel much freer.”* (Zainab, questionnaire)

Several participants (~ 10%) described how since treatment they had been able to pursue goals and interests that were not weight or shape-related (e.g. university studies, travelling).

*“[treatment] has changed my life and has got me back to university.”* (Emma, questionnaire)

*“Thanks to FREED I managed to do my A-levels and thus maintain my prospects. Without this service it is likely my life would have been very different”.* (Lucy, questionnaire)

Of those who ascribed subtle changes to treatment, or did not believe treatment had helped them, some participants (~ 2% of those providing questionnaire data) felt they had been discharged from treatment before change had been achieved, and they felt hopeless and abandoned.

*“I feel a sense of hopelessness. I was discharged a few months ago - because they felt there was nothing more they could do to help me, with my weight remaining the same, and my depression worsening.”* (Roisin, questionnaire)

More commonly, participants (~ 10%) identified that they came to treatment without any hope of recovery, and that FREED had instilled a sense of hope. Rather than feeling abandoned, they felt empowered and well-equipped to move forward in their recovery without a therapist.

*“The FREED team have given me hope that I can make a full recovery.”* (Victoria, questionnaire)

*“I still have a lot of work to do but I can see that I’m going somewhere now.”* (Chloe, questionnaire)

#### Treatment process

Five key themes relating to participants’ experiences of treatment process were identified in the data: i) rapid access to treatment; ii) knowledgeable and concerned clinicians; iii) building a support network; iv) life beyond the ED; v) becoming own therapist. These themes were primarily derived from data collected via interview.

##### Rapid access to treatment

About one-quarter of participants who provided questionnaire data identified that treatment had been provided quickly through FREED. Participants described this lack of wait positively and expressed positive attitudes towards the concept of “early intervention” more broadly.

*“Early intervention is imperative. I'm at my healthy BMI now and feel great, … just seven or eight months from being at my lowest weight, heart rate, mental state.”* (Georgia, questionnaire)

Many interviewed participants had anticipated a long wait for treatment upon referral to specialist services and described being pleasantly surprised or disbelieving when told they would shortly begin treatment. Several participants used words such as “lucky” or “fortunate” and reported feeling guilty or worried about others who did not receive treatment as quickly.

*“I'm eternally grateful for [rapid access to treatment]… [it’s] tinged with quite a bit of remorse I guess, for those people who weren't as fortunate.”* (Max, interview)

Participants reported beliefs that rapid access to treatment had been instrumental to their personal progress and/or was a positive thing more broadly, for several reasons.

*Prevented behaviours becoming more engrained*. Participants (~ 10%) expressed the belief that treatment had been provided at “just the right time” and believed that their difficult thoughts and behaviours would have become more engrained if they had to wait any longer for treatment.

*“It's so hard to get out of that rut, but the less time you spend there in the first place the easier it is.”* (Georgia, questionnaire)

Several interviewed participants saw early intervention as preventing their eating difficulties becoming long-running issues.

*“I'm like [to friends who are struggling] if you get help now you'll be fine, but if you wait it's going to be so hard, and you will never really be able to recover… it's something they will have to live with for the rest of their life.”* (Chloe, interview)

However, one interviewed participant expressed that - although they regarded the concept of early intervention positively - it did not happen early enough. At point of starting treatment, their difficult thoughts and behaviours were already engrained.

*“Maybe I wasn't the best person to use it because, although it's the first time I've properly been willing to go through [treatment], … I'd been dealing with it for quite a long time. I think that it needs quite newly-formed habits, that it might work a bit better because they're not as entrenched.”* (Gemma, interview)

*Prevented deterioration of physical health*. Participants (~ 10%) expressed the belief that their physical health would have deteriorated further if they had waited longer for treatment.

*“If I had not seen my therapist as quick as I did, my bulimia would have continued, and I'd be in a much worse medical situation than I am now.”* (Daisy, questionnaire)

Both questionnaire and interview data indicated participants’ beliefs that early intervention had been “life-saving” and believed they might not still be alive if they had not received care when they had.

*“I would definitely be dead by now if I hadn’t have received medical intervention*.” (Poppy, questionnaire)

Several participants described at interview how they had previously experienced long waiting lists for ED treatment and provided insight as to how that affected their physical health.

*“There was a very long waiting period to getting help and, in the meantime, … things were deteriorating pretty quickly…. five months is a long time to be malnourished for, so it's a big, big impact”.* (Max, interview)

*Striking whilst the iron is hot*. Several participants reported ambivalence and apprehensions about treatment when they were referred to specialist services. Participants believed rapid access to treatment had been important because they had limited time to reconsider their decision to get help.

*“Initially, the denial and shame made me rethink my decision to get professional help but having early intervention meant that I was quickly in therapy and able to explore my doubts there.”* (Zira, questionnaire)

Indeed, one interviewed participant described her previous experience of changing their mind about engaging with treatment whilst waiting for it to be provided.

*“It took a really long time.....by the time I did [get offered treatment], I kind of thought that...I was OK, and I didn't really want to go and I kind of just thought I can deal with this on my own.”* (Gemma, interview)

For several participants, ambivalence about receiving therapy was linked with beliefs that they were not “ill enough” to warrant treatment. Participants described that quick access to treatment validated their difficulties and facilitated the process of accepting the seriousness of their ED.

*“It felt like the NHS was saying we understand that you are sick, we care, and we want to help now, you don't have to live like this, and that got me to my first appointment.”* (Zira, questionnaire)

##### Knowledgeable and concerned clinicians

Several participants (10%) spoke positively of the professionals who had provided FREED treatment and identified them as an important contributor to the progress they had made.

*“Please just tell [therapist name] I am eternally grateful for her help. She's a diamond.”* (Noah, questionnaire)

Several participants elaborated at interview that they believed that clinicians were highly knowledgeable about EDs, which made them feel safe and understood.

*“I was more comfortable talking to the therapist at the eating disorder service…I think I just felt more kind of understood…I guess because they're used to talking to people…with similar problems, whereas GPs see everyone”.* (Sasha, interview)

Additionally, participants valued that clinicians expressed concern about the potential impact of their eating difficulties on physical health or achievement of life goals. Several participants described at interview how early appointments were characterised by a sense of urgency, emphasising that changes would need to be made early to prevent lasting damage.

*“I was given an emergency meal plan, and all these vitamins and minerals to get, because I'd been underweight for quite a long time… and they were concerned about malnutrition.”* (Christina, interview)

These expressions of concern and the need for immediate change facilitated the process of accepting the seriousness of their condition, enhancing motivation to change.

*“My therapist and the dietitian did give me a lot of information about…what malnutrition does to your brain…that did really help me to realise…I definitely don't want to be doing this to my brain.”* (Gaia, interview)

##### Building a support network

Several participants described at interview how they had been encouraged throughout treatment to share resources (e.g. psychoeducational material) with family and friends and been given opportunities to include their family or friends at various stages of the treatment process (e.g. bringing a friend/family member to their assessment or a therapy session). Some participants reflected upon this emphasis on family/friend involvement as a challenging aspect of treatment, often because of fears about being a burden to others and beliefs that they should “do it themselves”.

*“They came to one session, but I didn’t really want them to... I didn’t like it.”* (Aaliyah, interview)

Despite it being challenging, participants identified that they had been able to increase the involvement of their family and friends over the course of treatment. They reflected that building a support network in this way had been beneficial and important to the progress they had made.

*“The support provided to my family and myself was incredible and helped us all learn how to deal with my anorexia as a team.”* (Georgia, questionnaire)

##### Life beyond the eating disorder

Several participants identified at interview that treatment did not focus exclusively on their eating difficulties but looked at eating difficulties within the broader context of their life. They reported that the treatment felt tailored to their age-group.

*“It was really focused on… my age, the position I was in.”* (Annie, interview)

A minority of participants (2% of those providing questionnaire data) felt that their treatment was too focused on eating, with monitoring of weight in particular experienced as coercive and detrimental to health.

*“I was forced to weigh in every week which I don’t think was valid and made me even more ill.”* (Ruth, questionnaire)

For those participants who did feel their treatment was holistic, they identified that this focus had contributed to progress in several ways.

*Life getting in the way of treatment*. Several participants described at interview how instability or unpredictability in their broader life context (e.g. busy jobs with limited freedom to plan schedule, returning to the family home for summer holidays) made it more difficult to engage with treatment. Participants described how service flexibility (e.g. out-of-hours appointments; ease of rescheduling) was important to mitigate the impact of this instability on treatment.

*“If one week I couldn't make it wasn't a problem, we'd just arrange for next week. That's been a real benefit, just sort of working it in around a busy job.”* (Max, interview)

Some participants described finishing treatment because of a residence change (e.g. moving to and from the parental home in line with university terms) and expressed beliefs that such transitions had been well-managed.

*“I was transferred to services in Devon, and I was very lucky, because it came under a transfer of care, rather than a new referral.”* (Phoebe, interview)

However, several participants felt unclear as to how many sessions they were to receive and identified that knowing this would have been helpful for goal-setting.

*“I wasn't really aware until coming towards the end of my sessions….how long [treatment] was going to be… that would be nice because obviously… [you can set] your goals a bit more specifically if you've got a time-frame.”* (Max, interview)

*Focusing on future goals*. Participants (~ 10%) described how treatment focused on future goals and getting back to life without an ED. Several participants described how clinicians explicitly discussed the detrimental impact of eating difficulties on achievement of life goals (characterised by some as a “scare-talk”), which enhanced motivation to change.

*“In my first appointment, I was told that my plans to pursue a career in music and academia would be non-existent if my anorexia continued.”* (Hannah, questionnaire)

Many participants believed that treatment had been focused on helping them work towards goals related to life beyond their ED.

*“The good thing with this treatment compared to other treatments was that this had an end-goal... we had goals about becoming more independent, dealing with change, starting university.”* (Eleanor, interview)

Some participants described receiving support from clinicians regarding working towards future life goals (e.g. starting university; career decisions).

*“we did a lot of preparation during the summer months [for university], around forming relationships with people, eating lunch with people.”* (Annie, interview)

*“[my therapist] was trying to help me like get back in touch with the things that I like, and the things that make me “me” … I couldn’t even decide what job I wanted to, and I managed to decide I'd like to be a paramedic.”* (Olivia, interview)

##### Becoming your own therapist

Some participants identified tools that focused on helping them to monitor and self-manage their eating difficulties as beneficial (e.g. food diaries and worksheets to complete between sessions). A number of participants described how tools such as food diaries helped them to understand connections between environmental triggers and their emotions, thoughts, and behaviours, and feel more able to control their behaviour.

*“The food diary idea really helps you keep a close eye on what goes on and how you can progress.”* (Hayley, questionnaire)

Indeed, several participants suggested at interview that more homework would be welcome.

*“[I’d suggest] perhaps maybe more exercises or tasks to do in my own time, to kind of challenge how I was dealing with the things, or the new strategies that they'd given me.”* (Niamh, interview)

Participants described how such resources helped them feel empowered to move forward in their recovery without a therapist.

*“I genuinely feel I have the tools to beat my eating disorder without a professional now.”* (Iris, questionnaire)

*“I felt so supported all the way through, but also empowered to help myself.”* (Clara, questionnaire)

## Discussion

### Summary of findings

This is the first study to explore patients’ experiences of a developmentally-informed early intervention service model for EDs. Participants were emerging adults with first-episode, recent-onset EDs who received treatment through FREED. The majority of participants had found FREED treatment helpful and reported noticeable psychological and behavioural changes. Participants identified rapid access to treatment, knowledgeable and concerned clinicians, building a support network, focus on life beyond ED and becoming own therapist as key beneficial characteristics of the treatment received.

#### Psychological and behavioural change

Whilst most participants reported noticeable psychological and behavioural changes, there was variation in how such changes were conceptualised. Some participants described positive progress in terms of symptomatic improvement, whilst many participants described it in terms of being “freer” and more able to be themselves or being able to work towards goals that were not ED-related. Many participants acknowledged that they were not yet “recovered”, but that treatment had made them feel empowered to work towards recovery. Importantly, a small minority of participants believed that treatment had not been associated with change, in some cases because treatment was experienced as too brief. These findings both corroborate and enrich quantitative evidence from the broader FREED-Up study, which found that FREED patients experienced significant change in ED symptoms and individualised outcomes over the course of treatment [30].

##### Rapid access to treatment

This study found that emerging adults expressed positive attitudes about the concept of early intervention and believed it had been instrumental to improvement in their ED. This finding is consistent with previous research which has found positive attitudes towards early intervention amongst emerging adults with EDs and other diagnoses, and suggests that rapid access to treatment may be key to the improved clinical outcomes associated with FREED treatment [10, 26, 28, 39].

In particular, participants believed that rapid access to treatment had prevented their ED thoughts and behaviours becoming more engrained and their physical health from deteriorating further. This in turn made their difficulties easier to overcome. This understanding is compatible with theories of ED progression and illness stage models on which the rationale for early intervention service models for EDs are based [40]. It is also in keeping with findings within the broader FREED-Up study, with non-FREED patients more unwell at assessment than FREED patients (i.e. their condition has worsened whilst waiting for treatment) [28].

Participants reported that rapid access meant they received care before they had time to reconsider their decision to access treatment. This is a novel finding but is broadly consistent with findings within the broader FREED-Up cohort, which found that treatment uptake was higher in FREED than in non-FREED patients [29]. Findings from this study suggest that such increased treatment uptake may be due in part to the ability of early intervention to “strike whilst the iron is hot”. Indeed, previous research has identified that “motivation to change” at start of ED treatment is associated with treatment outcome [41], and it may be that increased motivation to change at start of treatment can also help to explain FREED’s clinical effectiveness.

This study also found that some participants perceived that early intervention, as delivered here, was “not early enough” for them, because they already had had symptoms for some time prior to specialist referral. This is perhaps not surprising, given the evidence that emerging adults tend to come to services with a longer duration of ED than other groups [11, 12, 42, 43]. However, this finding further highlights that efforts to increase early help-seeking will be key to maximise the effectiveness of early intervention [43].

##### Knowledgeable and concerned clinicians

Participants experienced clinicians as knowledgeable and concerned, and believed these characteristics were important facilitators of positive change. This finding aligns well with extensive evidence which suggests the important of therapeutic alliance to patient experience and treatment outcomes, particularly for younger patients and those with AN [44, 45]. It also corroborates previous research which has highlighted the importance of staff expertise to emerging adults’ treatment experiences, both in EDs and more broadly [10, 39]. The perceived value of clinicians expressing concern is interesting, given that this is consistent with the FREED model’s emphasis on encouraging early symptom change through psychoeducation [26]. This finding therefore provides further evidence that this core characteristic of the FREED model is valued by patients and may be a contributing factor to improved clinical outcomes.

##### Building a support network

Efforts to recruit friends and family into a support network during treatment were viewed as beneficial by participants, and as important to the progress they had made in treatment. This is consistent with research which has demonstrated the clinical effectiveness of family-based ED treatment adapted for “transition-aged youth” (i.e. emerging adults) [46]. However, many participants were ambivalent about the inclusion of family or friends in their treatment. This corroborates previous research which has found this is often a challenging area for services working with emerging adults with EDs and other diagnoses [39, 46, 47]. It is not surprising that this is a particularly pertinent concern during emerging adulthood, given that this life-stage is characterised by ongoing autonomy development and an “in-between” (childhood / adulthood) level of independence [48, 49]. Indeed, asserting self-reliance and independence from caregivers has been previously highlighted as a particular concern for emerging adults with EDs [9, 10, 43].

##### Life beyond the eating disorder

Many participants appreciated the focus on life beyond their ED in treatment, although a small minority believed treatment had been too focused on eating. In particular, participants reported instability in their lives, and that life sometimes “got in the way” of treatment. This is in keeping with existing evidence that emerging adulthood tends to be characterised by instability and a proliferation of life-events, which in turn have the potential to impact emerging adults’ help-seeking and treatment [9, 10, 37, 48]. Importantly, participants were pleased with the flexibility FREED service showed to counteract such instability (e.g. accommodating rescheduling, supporting with transition arrangements), and believed this contributed to positive outcomes.

Additionally, participants valued that FREED treatment was focused on working towards life goals. Emerging adulthood is a key time for psychosocial development, particularly identity development in multiple life domains (e.g. relationships; vocation; ideology) [50]. Furthermore, burgeoning research has identified the important interplay between ED aetiology /treatment response and ongoing developmental context, with concurrent identity development identified as particularly relevant [9, 10, 37, 51, 52].

##### Becoming your own therapist

Participants valued that treatment focused on them “becoming their own therapist”. This is perhaps not surprising, given that emerging adulthood is a key time for autonomy development and tends to be characterised by attitudes of self-reliance [48, 49]. Making the most of such attitudes (whilst also emphasising the importance of social support) may be key to the improved clinical outcomes associated with FREED treatment [10, 26, 28]. Interestingly, this finding aligns with existing qualitative literature exploring young peoples’ experiences of early intervention services for psychosis, suggesting this concept’s relevance across diagnoses. A recent meta-synthesis of all such studies found that participants spoke about how treatment through early intervention services helped them realise the importance of personal agency in their recovery journey [39].

### Strengths and limitations

Participants were drawn from a well-established and comprehensive cohort study. Data pertaining to key demographic and clinical characteristics were therefore available, and the sample is clearly characterized. Data were triangulated from two sources, which is likely to have increased the validity and comprehensiveness of the findings. Questionnaires allowed exploration of the experiences of a large cohort, whilst interviews offered the opportunity for experiences to be explored in greater depth.

Qualitative data regarding treatment experience were not collected from emerging adults who did not receive treatment through FREED. It is therefore not possible to make definitive statements about the extent to which the treatment experiences outlined in this paper were unique to FREED. Additionally, just 38% of eligible individuals opted to provide data for the study. Comparison of the characteristics of those who did and did not provide qualitative data indicates that these groups are broadly similar on a range of characteristics. However, the treatment completion rate was higher amongst those who provided qualitative data than amongst those who did not. It may be this sample is biased towards people who had more positive experiences of treatment.

Finally, whilst the researchers who conducted this study do not provide FREED treatment, their professional perspectives are “pro-FREED”. Efforts were made to bring this bias and its impact to awareness and mitigate it as far as was possible (e.g. by considering such biases within the analytic process).

### Future research

This study focused on those emerging adults who accepted the offer of and engaged with FREED treatment. Considering that 9% of emerging adults referred to FREED-UP could not be contacted after referral or did not attend their assessment appointment [29], future research might usefully consider the experiences of people who decide not to engage with treatment. Additionally, future quantitative research might focus on investigating the mechanisms of effect suggested by the present study (e.g. inclusion of measures of motivation to change, identity development).

### Clinical implications

This study provides further support for wide-scale implementation of early intervention and developmentally-informed models in ED care for emerging adults. From a patient perspective the key characteristics of this model appear not just to be rapid access to treatment but also the focus on meeting developmental needs. With regard to future development, it is clear early intervention-focused approaches should be accompanied by efforts to increase early help-seeking in order to maximise their effectiveness.

## Conclusion

Emerging adults with first-episode, recent-onset EDs who received treatment through FREED saw treatment as helpful and reported noticeable psychological and behavioural changes. Participants identified rapid access to treatment, knowledgeable and concerned clinicians, building a support network, focus on life beyond ED and becoming their own therapist as key beneficial characteristics of the treatment received. These findings provide further support for the value of adopting early intervention, developmentally-informed models in ED care.

## Data Availability

The datasets used and/or analysed during the current study are available from the corresponding author on reasonable request.

## Appendix

### Interview Topic Guide

1. Can you tell me about your experience of treatment within the FREED service?
  **Probes:**
  - Impact / change
2. Was there anything you found helpful during treatment?
3. Was there anything you didn’t like about treatment?
4. Do you have any suggestions for how we might improve the service?
5. Did the service feel sufficiently tailored to *your* needs?
  **Probes:**
  - How so?
6. What is your understanding of “early intervention”?
  **Probes:**
  - Importance? Why /why not?
  - Impact
7. Were your parents or friends involved in your treatment at all?
  **Probes:**
  - Who, how?
  - Thoughts / feelings
8. Did you have to transition to another service during your treatment?
  **Probes:**
  - Experience
9. You are in a time of life when you are learning to do lots of things, becoming more independent and figuring out who you are. Did treatment help with things beyond ED or was it mostly focused on your ED?
  **Probes:**
  - Developing identity e.g. career goals
  - Skills for independent living

## Abbreviations

AEDS: Adult eating disorder services
AN: Anorexia nervosa
BED: Binge-eating disorder
BN: Bulimia nervosa
CAEDS: Child and adolescent eating disorder services
EDs: Eating disorders
FREED: First Episode Rapid Early Intervention for Eating Disorders
OSFED: Other specified feeding and eating disorders
UK: United Kingdom
